# Using a discrete choice experiment to elicit patients’ preferences and willingness-to-pay for knee osteoarthritis treatments in Thailand

**DOI:** 10.1038/s41598-023-39264-6

**Published:** 2023-07-27

**Authors:** Parnnaphat Luksameesate, Aree Tanavalee, Surachat Ngorsuraches, Suthira Taychakhoonavudh

**Affiliations:** 1 Present Address: Department of Social and Administrative Pharmacy, Faculty of Pharmaceutical Sciences, Chulalongkorn University, Bangkok, Thailand; 2 Present Address: Department of Orthopaedics, Faculty of Medicine, Chulalongkorn University, Bangkok, Thailand; 3 Present Address: Department of Health Outcomes Research and Policy, Harrison School of Pharmacy, Auburn University, Alabama, USA

**Keywords:** Signs and symptoms, Health care, Diagnosis, Disease prevention, Geriatrics

## Abstract

Osteoarthritis is the most common type of joint disease among elderly patients around the world. In response to the need for patient-centered care, patients’ and physicians’ preferences for knee osteoarthritis treatments have been studied in multiple countries, but not in Thailand. The objective of this study was to investigate Thai patients’ preferences and their willingness to pay (WTP) for knee osteoarthritis treatments by using a discrete choice experiment (DCE). Six knee osteoarthritis treatment attributes, including pain relief, delayed disease progression, gastrointestinal side effects, kidney side effects, cardiovascular side effects, and cost, were used to develop a paper-based, DCE questionnaire survey. Patients with knee osteoarthritis, who were at least 18 years old and who provided written informed consent, were recruited from the orthopedic department in a tertiary care hospital in Thailand via convenience sampling. The conditional logit model was used to determine patients’ preferences and WTP. The Institutional Review Board at Chulalongkorn University approved this study before it started. A total of 232 patients were collected and analyzed in this study. Patients preferred treatments with a higher efficacy (pain relief and delayed disease progression), a lower probability of side effects (gastrointestinal, kidney, and cardiovascular side effects), and a lower cost. Regarding efficacy and side effects, the patients weighted the importance of a 1% change in cardiovascular side effects (− 0.08) more heavily than 1% changes in kidney (− 0.07) and gastrointestinal (− 0.02) side effects, delayed disease progression (0.02), and pain relief (0.01). Patients were willing to pay 29.56 Thai Baht (THB) and 41.84 THB per month for every 1% increase in pain relief and delayed disease progression, respectively. Conversely, patients were willing to pay 52.04 THB, 145.18 THB and 164.23 THB per month for every 1% decrease in gastrointestinal, kidney, and cardiovascular side effects, respectively. In conclusion, pain relief, delayed disease progression, gastrointestinal side effects, kidney side effects, cardiovascular side effects, and the cost of treatment were significant factors among patients undergoing knee osteoarthritis treatment. Additionally, patients had a higher WTP for delayed disease progression than pain relief and a higher WTP for a reduced probability of cardiovascular side effects than gastrointestinal and kidney side effects. These findings could be used to support treatment decisions for knee osteoarthritis patients in Thailand.

## Introduction

Osteoarthritis (OA) is the most common type of joint disease in elderly individuals. Signs and symptoms of this disease include pain, stiffness, tenderness and swelling of the hand, wrist, or knee joint areas. The World Health Organization (WHO) reported the global prevalence of OA in the *Chronic Diseases and Health Promotion*; it was found that 9.6% of men and 18.0% of women over 60 years old have asymptomatic OA^1^. The International League of Associations for Rheumatology (ILAR) and the Community Oriented Program for the Control of Rheumatic Diseases (COPCORD), which has studied the prevalence of various rheumatic diseases in Thailand and Asian countries, found that 3.7–39.2% of people in those countries had knee OA or chronic knee pain^2,3^. Another epidemiological study showed that the prevalence of knee OA in elderly Thai patients ranged from 34.5 to 45.6%, with more than half of these patients having pain in both knees^4^. 

Currently, several treatments are available for knee OA disease, including weight reduction, exercise, acupuncture, acetaminophen, nonsteroidal anti-inflammatory drugs (NSAIDs), symptomatic slow-acting drugs for osteoarthritis (SYSADOA), intra-articular corticosteroid and surgery^5^. NSAIDs, including diclofenac and etoricoxib, are the most commonly used treatments for knee OA because of their good efficacy. However, some side effects are frequently found, including gastrointestinal (GI), cardiovascular (CV) and renovascular systems^6^. Glucosamine sulfate is one of the treatment choices for mild to moderate knee OA with a good safety profile. For efficacy, glucosamine had been unclear in the past due to the mixed results from the various formulations and its impact on clinical outcomes^7^. In 2005, a Cochrane systematic review of 25 randomized controlled trials yielded inconclusive results regarding the benefits of glucosamine for pain relief^8^. Remarkably, subgroup analyses showed that only crystalline glucosamine sulfate was significantly more beneficial than placebo in terms of pain relief and functional improvement. Subsequent studies focused on the results from the different formulations of glucosamine. Recently, many studies have consistently demonstrated that crystalline glucosamine sulfate showed superior benefits over other formulations in terms of pain and functional improvement, delayed disease progression and reduced use of long-term NSAIDs^9–14^. With the results of cost-effectiveness, one study compared glucosamine to paracetamol and found that glucosamine was cost-effective and dominant compared with paracetamol and placebo^15^. Another study found that glucosamine presented a higher level of clinical effectiveness than current care for the treatment of knee OA but did not clearly demonstrate cost-effectiveness, which was related to the magnitude and duration of quality of life gain^16^.

In Thailand, some knee OA treatments, including diclofenac, are listed in the National List of Essential Medicines (NLEM), which is used as a reimbursement list for all three major health insurance schemes, including the Civil Servant Medical Benefit Scheme (CSMBS), Universal Coverage Scheme (UCS), and Social Security Scheme (SSS). However, some treatments, including glucosamine sulfate, were restricted for reimbursement in the USS, SSS and CSMBS. The key reimbursement considerations for listing drugs on NLEM consist of safety and efficacy, the cost index, the cost-effectiveness analysis, and budget impact analysis results^17,18^. Two studies conducted an economic evaluation of glucosamine in Thailand. One study reported that glucosamine was not cost-effective for OA treatment at Thailand’s willingness to pay (WTP) threshold^19^. However, this study was performed under the Thai Ministry of Public Health perspective, which left outpatient as well as societal perspectives. Inconsistency results in another study, under a societal perspective, showed that the addition of crystalline glucosamine sulfate into standard treatment was cost-effective at the WTP threshold in Thailand^20^. Additionally, early adoption of crystalline glucosamine sulfate would be less costly and more effective than delayed treatment or the use of standard treatment alone.

While the NLEM has a major impact on patient access to knee OA treatments, patients’ preferences for these treatments have not been included in these considerations. Without clinicians’ and policy-makers’ understanding of the patient’s perspectives on the treatments, patients may not adhere to the treatments and eventually experience poor health outcomes^21^. However, studies that rigorously examined patients’ preferences for knee OA treatments are available only in the United States, Australia and France. One study compared preferences for NSAIDs, opioids, glucosamine/chondroitin and capsaicin, considering only the risk of GI ulcers^22^. The results showed that patients were willing to receive the treatment with a lower risk of adverse effects. The second study compared preferences for oral prescription medication, steroid injection and viscosupplementation^23^. They concluded that patients preferred a longer-lasting pain relief treatment. However, neither study included CV and kidney side effects, even though these side effects are among the frequently found side effects of NSAIDs. Another study compared preferences for NSAIDs, glucosamine and acetaminophen which were roughly divided risk into three levels^24^. Both cardiovascular and kidney side effects were included in the analysis but combined into one attribute, resulting in separating out the impact of each side effect on patient preference. Additionally, little is known about the preference of knee OA patients in the Thai population.

The use of stated preference techniques in the form of conjoint analysis (CA) has grown for eliciting preferences. One of the commonly used CAs is the discrete choice experiment (DCE), which has increasingly been applied in the health care sector to measure preferences and quantify the relative importance of the benefits and risks of different health care products and programs^25^. A DCE involves the generation of hypothetical choice sets incorporating multiple characteristics. Respondents are asked to choose among different choice sets. Currently, several published studies have used DCE as a tool to investigate patients’ preferences for OA treatment among other CA techniques since it could identify attributes significantly influencing preferences^25^. In addition to low cognitive complexity, it is easy to answer and use a small sample size to estimate WTP. With these benefits, the DCE method was the most appropriate tool to elicit the preferences of elderly patients in one tertiary care hospital included in the study. The objective of this study is therefore to investigate patients’ preferences and their WTP for knee OA treatments in Thailand by using DCE.

## Materials and methods

This study used a cross-sectional, DCE questionnaire survey design to determine patients’ preferences for knee OA treatments. A DCE has been used to examine stated preference by asking patients to consider various choice sets that include multiple alternatives described by study attributes and their alternatives. Based on random utility theory (RUT), a utility model is then developed to determine the relative importance of treatment attributes.

### Attribute and level elicitation

To identify the key attributes of knee OA treatments, literature reviews were conducted to derive an initial list of attributes and the focus group was performed to prioritize and determine the key attributes as recommended by Helter and Boehler^26^. From the literature, there were 35 attributes divided into three main groups of knee OA treatments including efficacy (e.g., pain relief, stiffness, physical function, delayed disease progression, onset of action), side effects (e.g., GI, CV, hepatic, kidney, skin ulceration, anemia) and costs^9,11,13,15,27–32^. These attributes were then used as a guide for focus group discussion to elicit the important treatment attributes. A total of seven knee OA patients participated in the focus group through consultation with the orthopedist. The focus group members consisted of six females and one male. Of these patients, the average age was 66 years and the average number of years after OA diagnosis was 5.71 years. The average pain rating score for their knee pain was 4 points out of 10. All of them previously used glucosamine sulfate and NSAIDs as knee OA medications. We initiated the focus group discussion with an overview of the topic, the objective of the study, the ground rules, and the duration of the discussion. A total of 35 attributes from the literature reviews were then introduced to the members for their opinions on the significance and relevance regarding the OA medication and the new attributes were also allowed for discussion to add to the list (if any). The group members were encouraged to communicate and exchange ideas on each other’s experiences. At the end of the discussion, the six most important attributes were summarized to ensure correct interpretation and allow the group members to elaborate their points further.

Based on the group consensus, the following six attributes were examined: pain relief, delayed disease progression, GI side effects, kidney side effects, CV side effects, and the cost of treatment per month. These attributes were varied across three plausible levels derived from the clinical literature. The range of levels for each attribute was designed to present the efficacy outcomes and side effects of knee OA treatments that could be seen in clinical trials or clinical practice. The highest and lowest levels of pain relief were extracted from the efficacy of steroid injection and acetaminophen respectively^33–36^. Glucosamine sulfate was used as the treatment that could reduce the risk of disease progression by 50% while other treatments were not reported^37^. The maximum level of probability of GI, kidney, and CV side effect attributes was derived from the side effects of NSAIDs^38–41^. The levels of the cost attribute were obtained from the Drug and Medical Supply Information Center (DMSIC), Ministry of Public Health^42^. This study assumed that patients received treatments with the maximum doses allowed for knee OA—acetaminophen (3000 mg/day), diclofenac (150 mg/day), etoricoxib (60 mg/day), glucosamine (1500 mg/day), and a triamcinolone acetonide injection (40 mg/every 3 months)^43,44^. The average price of all generic names of each treatment was used to calculate the cost per month. The attributes and their levels are shown in Table 1.

**Table 1:** Study attributes and their levels.

Attribute	Level	References
Pain relief	30%, 50%, 70%	^33–36^
Delayed disease progression	0%, 25%, 50%	^37^
Probability of gastrointestinal side effects	0%, 35%, 70%	^38^
Probability of kidney side effects	0%, 13%, 26%	^39^
Probability of cardiovascular side effects	0%, 12%, 24%	^40,41^
Cost per month	0 Baht, 600 Baht, 1200 Baht	^42^

### Questionnaire survey development

**Figure 1:**
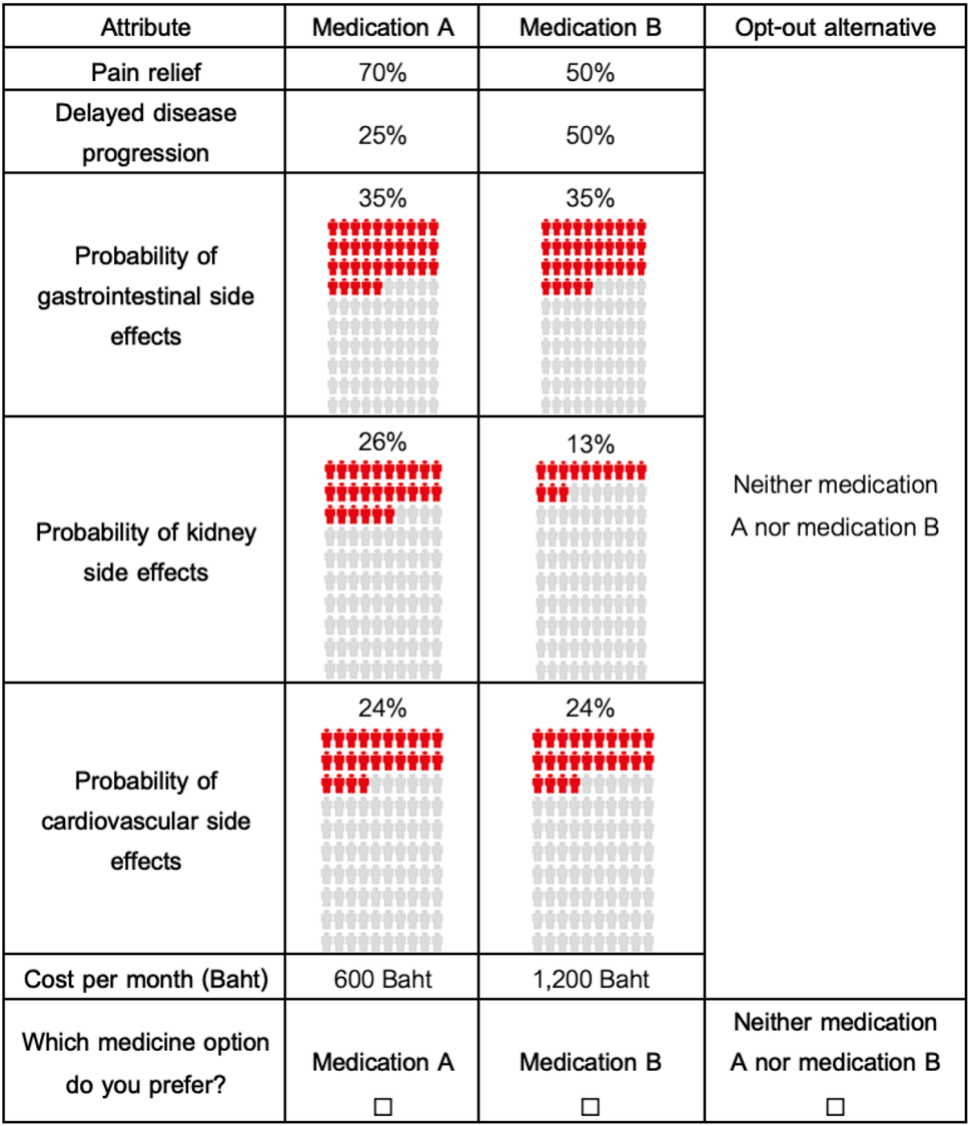
A choice set example.

A paper-based, self-administered questionnaire survey was developed. The total number of possible combinations of the attributes and levels in this study was 729 (3 × 3 × 3 × 3 × 3 × 3 × 3). Since it was not possible to include all combinations in a questionnaire survey, an orthogonal design (a fractional factorial design) was used to randomly draw a subset of all combinations by using Ngene software (V.1.2.1). A total of 36 choice sets were generated and then divided into six blocks. Each questionnaire consisted of six choice sets from each block. Therefore, there were six questionnaire versions in this study. Each choice set contained two unlabeled, hypothetical OA treatment alternatives and one opt-out alternative, as illustrated in Fig. 1. Another choice set, which contained a dominant alternative with the highest efficacy, lowest side effects, and lowest cost, was added to every version for a validity check. Patients were asked to choose a preferred alternative from all choice sets. Questions on patient characteristics and OA-related experiences were included. Three experts, including an orthopedist, a pharmacy faculty member and a clinical pharmacist, were asked to check the content validity of the questionnaire survey. The survey was piloted with 15 knee OA patients. No major problem was found during the pilot test.

### Patient recruitment

Patients with knee OA who were 18 years old or above, treated and presented in the orthopedic department of a tertiary care hospital (Chulalongkorn Hospital), were conveniently sampled by the researcher. Patients with mental disorders or a knee replacement planned within three months were excluded. The recruitment questions asked patients (i) had they been diagnosed with OA, (ii) what was the location of arthritis, (iii) did they plan knee replacement within three months, and (iv) were they willing to participate in the study. All patients who passed the recruitment questions and agreed to provide written informed consent were included in the study. Patients who participated in the focus group were excluded from participating in this main study.

### Sample size

The sample size calculation differed substantially among the DCE studies with a range of smaller than 100 to larger than 1000 patients^45^. However, one of the following rules of thumb proposed by Johnson and Orme^45^ was increasingly used to estimate the minimum sample size requirement. The sample size was calculated from the Formula N > 500c/(t × a), where t is the number of choice tasks, a is the number of alternatives, and c is the number of analysis cells which was equal to the largest number of levels for any of the attributes. At least 125 patients were needed in the study.

### Data collection

Each patient who passed the recruitment questions was invited to respond to one random version of the paper-based questionnaire survey and received a brief description of the study objective and an explanation of the attributes and levels. The questionnaire comprised three sections and was self-reported. Patient characteristics, including age, gender, weight, height, marital status, education level, health insurance, total monthly personal income, total monthly household income for all family members, and disease history, were requested to be answered in the first section of the questionnaire. In the second section, patients were asked to indicate knee OA-related experiences, including the number of years of OA experience, the severity of OA symptoms, and medication history. In this section, a visual analog scale was designed to document the disease-related symptom severity in OA patients. For the final section, six choice sets were presented to evaluate knee OA treatment preferences. For each choice set, patients were assessed using the following question: “What medicine option do you prefer when receiving treatment for their OA?” Patients could choose among three alternatives: two medications with different efficacy, side-effect and cost profiles, or opt-out alternative. To check and determine the understanding, one choice set, which contained the superiority on all attribute levels to the comparator scenario, was added to every version of the questionnaires. This dominant scenario should rationally be the chosen option. If they did not answer the dominant scenario in this validity check, their data were removed from the analysis. A token of appreciation was given to the patients after they finished.

We assessed patient preference based on the utilities extracted from the DCE questionnaire using the conditional logit model. The patient values were evaluated from the following treatment characteristics: pain relief, delayed disease progression, GI side effects, kidney side effects, CV side effects, and the cost of treatment per month.

### Data analysis

Only responses from the patients who answered all questions completely were included in the data analyses. All data analyses were conducted by using StataIC 16. The patient characteristics and OA-related experiences were descriptively analyzed. Based on the RUT, the utility (U) for each patient (i) selecting an alternative (j) was developed^46^:

$$\begin{gathered} {\text{U}}_{{{\text{ij}}}} = \beta_{0} + \beta_{1} {\text{pain}}\;{\text{relief}}_{{\text{j}}} + \beta_{2} {\text{delayed}}\;{\text{disease}}\;{\text{progression}}_{{\text{j}}} + \beta_{3} {\text{GI}}\;{\text{side}}\;{\text{effects}}_{{\text{j}}} \hfill \\ \quad \quad \; + \beta_{4} {\text{kidney}}\;{\text{side}}\,{\text{effects}}_{{\text{j}}} + \beta_{5} {\text{CV}}\;{\text{side}}\,{\text{effects}}_{{\text{j}}} + \beta_{6} {\text{cost}}_{{\text{j}}} + \varepsilon_{{{\text{ij}}}} \hfill \\ \end{gathered}$$

where β_0_ is a constant, β_1_ to β_6_ are the coefficients of pain relief, delayed disease progression, GI side effects, kidney side effects, CV side effects, and cost, respectively, and ℇ is the error term. The statistical significance level was set at 0.05. The conditional logit model was developed to estimate the utility model. The value of each coefficient reflected the relative importance of each attribute. The sign represents whether the attribute had a positive effect or a negative effect on utility, as compared with the base level.

WTPs for each attribute was calculated as the ratio between the coefficient of each attribute and the coefficient of the cost attribute^47^. This parameter indicated how much patients were willing to pay for a one-unit change in each attribute. The Delta method was used to determine 95% confidence intervals of WTPs^48^.

### Ethics approval and consent to participate

The Institutional Review Board at Chulalongkorn University approved this study before it started (IRB No. 468/62). This study was conducted in accordance with the Declaration of Helsinki and the Principles in the Belmont Report. Informed consent was obtained from all patients.

## Results

A total of 237 patients with knee OA provided complete responses to the entire questionnaire. However, five of them (2.11%) were removed because they did not choose a dominant scenario in the validity choice set. The remaining 232 (97.89%) were included in the analyses. Table 2 shows the descriptive characteristics of the patients. The average age of the patients in this study was 68 years, and their average Body Mass Index (BMI) was 25.34 kg/m^2^. The majority of the patients were females (85.78%), married (62.07%), and unemployed (80.17%). Most of them reported that they had a bachelor’s degree or higher (63.36%). Approximately 58% of them used the CSMBS as their health insurance. The average individual monthly income was 28,631.25 Thai Baht (THB), and the average household monthly income was 72,732.11 THB. For the clinical characteristics of OA, the average number of years after OA diagnosis was 6.32 years. The average pain rating score for their knee pain was 4.30 points out of 10. The majority of them previously used glucosamine (86.64%) and NSAIDS (73.28%) as OA medications.

**Table 2:** Patient characteristics and OA-related experiences (N = 232).

Variables	
Age, years, mean (SD)	67.91 (7.22)
Female, N (%)	199 (85.78%)
BMI, kg/m^2^, mean (SD)	25.34 (4.03)
Marital status, N (%)	
Married	144 (62.07%)
Single	49 (21.12%)
Widowed/divorced/separated	39 (16.81%)
Education level, N (%)	
Elementary school or lower	27 (11.64%)
Junior high school	11 (4.74%)
High school	35 (15.10%)
Diploma	12 (5.17%)
Bachelor’s degree or higher	147 (63.36%)
Occupation, N (%)	
No occupation/retired from employment	186 (80.17%)
Employed	17 (7.33%)
Civil servant	18 (7.76%)
Own business	9 (3.88%)
Other	2 (0.86%)
Health insurance, N (%)	
Civil servant medical benefit scheme	135 (58.19%)
Social security scheme	7 (3.02%)
Universal coverage scheme	7 (3.02%)
Private insurance	3 (1.30%)
Self-pay	27 (11.64%)
Other	53 (22.85%)
Monthly income, mean (SD)	
Individual	28,631.25 (27,551.34)
Household	72,732.11 (78,288.56)
Number of years since diagnosed with OA, mean (SD)	6.32 (6.06)
Pain score, mean (SD)	4.30 (2.57)
Previous peptic ulcer or other GI diseases, N (%)	38 (16.38%)
Co-morbidity, N (%)	
Heart disease	11 (4.74%)
Kidney disease	4 (1.72%)
Medication history, N (%)	
Analgesic creams	146 (62.93%)
NSAIDs	170 (73.28%)
Glucosamine	201 (86.64%)
Steroid injection	42 (18.10%)
Hyaluronic acid	48 (20.69%)

### Patients’ preferences

Table 3 shows the results of the conditional logit model. Of the 232 patients, 221 (95.26%) chose either medication A or medication B. All coefficients’ signs were logical, and they were statistically significant (*p* < 0.001). The positive signs of pain relief and delayed disease progression indicated that the patients in this study preferred knee OA treatments with higher efficacy for pain reduction and a delayed rate of joint degeneration. The negative signs of GI side effects, kidney side effects, CV side effects, and cost attributes indicated that the patients preferred a lower chance of having side effects from the treatments and lower treatment costs. Among the efficacy and side effect attributes of the OA treatments, the patients gave a higher weight to the importance of a 1% change in CV side effects (− 0.08) than a 1% change in kidney (− 0.07) and GI (− 0.02) side effects, delayed disease progression (0.02), and pain relief (0.01). In summary, these results showed that the patients preferred treatments with higher efficacy, fewer side effects, and lower cost. In addition, the patients were more concerned about the risks of having side effects than the efficacy of the medicine, as indicated by the relative size of the coefficient attached to this attribute.

**Table 3:** Conditional logit model results.

Attribute	β coefficient	Standard error	*p* value
Pain relief	0.01462	0.00334	< 0.001
Delayed disease progression	0.02069	0.00278	< 0.001
Probability of gastrointestinal side effects	− 0.02574	0.00243	< 0.001
Probability of kidney side effects	− 0.07180	0.00534	< 0.001
Probability of cardiovascular side effects	− 0.08122	0.00617	< 0.001
Cost per month	− 0.00049	0.00012	< 0.001

Log-likelihood = − 602.81629, Pseudo-R^2^ = 0.3372.

### Patients’ WTPs

Table 4 shows the WTP for each attribute. The patients were willing to pay 29.56 THB and 41.84 THB per month for every 1% increase in the pain relief and delayed disease progression efficacies of the knee OA treatments, respectively. On the other hand, the patients were willing to pay 52.04 THB, 145.18 THB and 164.23 THB per month for every 1% decrease in GI side effects, kidney side effects, and CV side effects of the treatments, respectively.

**Table 4:** Patients’ WTP values for each attribute.

Attribute	Average WTP per month (Thai Baht)	95% confidence interval
Pain relief	29.56	10.19 to 48.94
Delayed disease progression	41.84	22.18 to 61.51
Probability of gastrointestinal side effects	− 52.04	(− 75.57) to (− 28.51)
Probability of kidney side effects	− 145.18	(− 213.44) to (− 76.92)
Probability of cardiovascular side effects	− 164.23	(− 241.66) to (− 86.79)

## Discussion

Our results showed that pain relief, delayed disease progression, GI side effects, kidney side effects, CV side effects, and cost were significantly important attributes when the patients chose knee OA treatments. Intuitively, the patients preferred treatments with higher efficacy, fewer side effects, and lower cost. While the patients focused on the pain relief and delayed disease progression efficacies of the OA treatments, they had concerns about the GI, kidney, and CV side effects of the treatments. In other words, they traded off between the benefits and risks when they chose the treatments. In addition, they also considered the amount of money that they needed to pay per month for knee OA treatments.

However, the importance of pain relief, delayed disease progression, GI side effects, kidney side effects, CV side effects, and cost for the patients varied. Our findings were consistent with three previous studies^22,24,49^, which concluded that patients with knee OA placed more importance on the lower risk of adverse effects than on the efficacy, such as reduced pain. Hauber et al.^49^ found that patients attached greater importance to eliminating the risks of adverse events than to reducing pain. Fraenkel et al.^22^ concluded that the risk of common adverse effects, e.g., GI ulcers, had the largest impact on patients’ preferences for knee OA treatments. It is noteworthy that CV side effects were not included in this study. Laba et al.^24^ found that delayed disease progression, side effects, out-of-pocket costs, and the schedule of treatment had impacts on the patient’s continuation of OA treatments. These side effects included high blood pressure and heart, kidney, and/or liver problems. In contrast, the other two studies’ findings were different^23,50^. Postnett et al.^23^ concluded that the amount of copayment and the duration of pain relief had the largest impacts on patients’ preferences for knee OA treatments, while Ratcliffe et al. found that the level of joint aches, the level of physical mobility, and the risk of serious side effects were significant attributes for OA patients^50^. One of the reasons was that these studies included different treatment attributes. Another reason would be that the patients in these studies were different in terms of demographic characteristics and preferences. For instance, our study tended to include older patients with knee OA. Therefore, their concerns about the side effects of OA treatments might be different.

The findings showed that the delay in disease progression was more important to the patients than the pain relief. The reason might be that the patients would like to delay the time when they needed knee surgery which could incur higher costs and risks. Among the side effects, it was intuitive that the patients weighed the CV and kidney side effects higher than the GI side effect since the GI side effects tended to be milder and easily managed.

We obtained the attribute levels of knee OA treatments including diclofenac, etoricoxib and glucosamine sulfate from published literature^33–35,37,38,51–53^ to calculate the WTP. We found that patients were willing to pay for glucosamine sulfate 2226 THB per month, while both diclofenac and etoricoxib were less preferred than the baseline. Interestingly, the WTP for glucosamine sulfate from this study was seven times higher than its generic current price (approximately 320 THB for one month) and one times higher than its original current price (approximately 1100 THB for one month) in Thailand. Conversely, diclofenac and etoricoxib were extremely low compared to their current price (approximately 22 THB and 460 THB for one month, respectively). These costs were obtained from the Drug and Medical Supply Information Center, Ministry of Public Health. The interpretation was that patients perceived the benefits of glucosamine sulfate more than its cost, while they perceived the benefits of diclofenac and etoricoxib less than their cost. The reason should be that glucosamine sulfate retarded the progression of the disease and had a good safety profile. Diclofenac and etoricoxib, NSAIDs, had good efficacy for the relief of pain, but they might have a problem with gastrointestinal, cardiovascular and renovascular systems. Although patients preferred the benefit of glucosamine sulfate, they were still restricted from accessing it because they had to meet all reimbursement criteria before starting treatment. The results of this study could emphasize the inclusion of the patient perspective in the development of reimbursement criteria.

Our study had some limitations. First, the study did not have a sufficient sample size to examine preference heterogeneity. It is possible that patients with different characteristics and OA-related experiences might prefer different knee OA treatments. However, the study findings would guide or encourage discussion among patients with knee OA treatments, their health care providers, and policy-makers when they make treatment decisions. Subsequently, the treatment decisions would be tailored for each individual patient. Second, this study collected data from only one tertiary care hospital. The majority of patients were well educated and had middle to high incomes. Therefore, the findings might not be generalized to other patient populations. Last, the limited number of attributes in this study might not completely explain patients’ preferences for knee OA treatments. It is possible that other treatment attributes might be significantly important to the patients. They should be examined further. Nevertheless, this study conducted a literature review and confirmed with patients and clinical experts to carefully select the attributes. They should include the majority of the important attributes.

## Conclusion

In conclusion, this study found that Thai patients focused on pain relief, delayed disease progression, GI side effects, kidney side effects, CV side effects, and cost when they chose knee OA treatments. For efficacy, the patients’ preferences weighed delayed disease progression higher than pain relief. Among all study side effects, CV side effects affected most of their preferences. Additionally, they valued net benefits for glucosamine sulfate but not for diclofenac and etoricoxib. The findings suggest that they are willing to pay more for glucosamine sulfate than diclofenac and etoricoxib. Our observations are intended to provide empirical evidence for physicians and regulators during the assessment of medication prescriptions, the development of practical guidelines, and the consideration of OA treatment selection into the National List of Essential Medicines.

## Supplementary Information

Supplementary Information.

## Data Availability

All data generated or analyzed during this study are included in this published article and its supplementary information files.
