# Supplementary material for: Using a discrete choice experiment to elicit patients’ preferences and willingness-to-pay for knee osteoarthritis treatments in Thailand
Source: Sci Rep. 2023 Jul 27;13:12154. doi: 10.1038/s41598-023-39264-6 (PMC10374609; doi:10.1038/s41598-023-39264-6)
Supplement: Supplementary file 1 — Supplementary Information. [file 41598_2023_39264_MOESM1_ESM.docx]

# **S1 Table. Orthogonal array of attribute level choice sets**

| **Question** | **Medicine A** | | | | | | **Medicine B** | | | | | |
| --- | --- | --- | --- | --- | --- | --- | --- | --- | --- | --- | --- | --- |
|  | **Pain relief** | **Slow disease progression** | **GI side effect** | **Kidney side effect** | **CV side effect** | **Cost** | **Pain relief** | **Slow disease progression** | **GI side effect** | **Kidney side effect** | **CV side effect** | **Cost** |
| **1** | 70 | 25 | 35 | 26 | 24 | 600 | 50 | 50 | 35 | 13 | 24 | 1,200 |
| **2** | 50 | 25 | 35 | 26 | 24 | 1,200 | 70 | 25 | 70 | 26 | 12 | 600 |
| **3** | 30 | 50 | 70 | 0 | 0 | 1,200 | 70 | 0 | 70 | 26 | 0 | 0 |
| **4** | 70 | 50 | 70 | 0 | 0 | 0 | 30 | 50 | 0 | 0 | 24 | 1,200 |
| **5** | 50 | 0 | 0 | 13 | 12 | 0 | 30 | 25 | 0 | 0 | 12 | 600 |
| **6** | 30 | 0 | 0 | 13 | 12 | 600 | 50 | 0 | 35 | 13 | 0 | 0 |
| **7** | 30 | 25 | 70 | 13 | 24 | 0 | 70 | 50 | 35 | 0 | 12 | 0 |
| **8** | 70 | 50 | 35 | 26 | 12 | 1,200 | 30 | 0 | 35 | 0 | 12 | 0 |
| **9** | 50 | 50 | 0 | 26 | 0 | 600 | 30 | 0 | 70 | 13 | 24 | 600 |
| **10** | 30 | 0 | 70 | 0 | 24 | 0 | 50 | 25 | 70 | 13 | 24 | 600 |
| **11** | 70 | 0 | 35 | 0 | 12 | 1,200 | 50 | 25 | 0 | 26 | 0 | 1,200 |
| **12** | 50 | 25 | 0 | 13 | 0 | 600 | 70 | 50 | 0 | 26 | 0 | 1,200 |
| **13** | 50 | 25 | 70 | 26 | 12 | 0 | 50 | 0 | 70 | 0 | 0 | 1,200 |
| **14** | 30 | 50 | 35 | 13 | 24 | 600 | 30 | 25 | 70 | 0 | 0 | 1,200 |
| **15** | 70 | 50 | 0 | 0 | 24 | 600 | 70 | 25 | 0 | 13 | 12 | 0 |
| **16** | 50 | 0 | 70 | 26 | 0 | 1,200 | 50 | 50 | 0 | 13 | 12 | 0 |
| **17** | 30 | 0 | 35 | 13 | 0 | 1,200 | 30 | 50 | 35 | 26 | 24 | 600 |
| **18** | 70 | 25 | 0 | 0 | 12 | 0 | 70 | 0 | 35 | 26 | 24 | 600 |
| **19** | 70 | 50 | 70 | 13 | 12 | 600 | 50 | 50 | 70 | 26 | 12 | 600 |
| **20** | 50 | 50 | 70 | 13 | 12 | 1,200 | 70 | 25 | 35 | 13 | 24 | 1,200 |
| **21** | 30 | 0 | 0 | 26 | 24 | 1,200 | 70 | 0 | 0 | 0 | 24 | 1,200 |
| **22** | 70 | 0 | 0 | 26 | 24 | 0 | 30 | 50 | 70 | 26 | 0 | 0 |
| **23** | 50 | 25 | 35 | 0 | 0 | 0 | 30 | 25 | 35 | 13 | 0 | 0 |
| **24** | 30 | 25 | 35 | 0 | 0 | 600 | 50 | 0 | 0 | 0 | 12 | 600 |
| **25** | 70 | 25 | 70 | 13 | 24 | 1,200 | 30 | 0 | 0 | 13 | 0 | 600 |
| **26** | 30 | 50 | 35 | 26 | 12 | 0 | 70 | 50 | 0 | 13 | 0 | 600 |
| **27** | 30 | 50 | 0 | 26 | 0 | 0 | 50 | 25 | 35 | 26 | 12 | 1,200 |
| **28** | 50 | 0 | 70 | 0 | 24 | 600 | 30 | 0 | 35 | 26 | 12 | 1,200 |
| **29** | 50 | 0 | 35 | 0 | 12 | 600 | 70 | 50 | 70 | 0 | 24 | 0 |
| **30** | 70 | 25 | 0 | 13 | 0 | 1,200 | 50 | 25 | 70 | 0 | 24 | 0 |
| **31** | 30 | 25 | 70 | 26 | 12 | 600 | 30 | 25 | 0 | 26 | 24 | 0 |
| **32** | 50 | 50 | 35 | 13 | 24 | 0 | 50 | 0 | 0 | 26 | 24 | 0 |
| **33** | 50 | 50 | 0 | 0 | 24 | 1,200 | 50 | 50 | 35 | 0 | 0 | 600 |
| **34** | 70 | 0 | 70 | 26 | 0 | 600 | 70 | 25 | 35 | 0 | 0 | 600 |
| **35** | 70 | 0 | 35 | 13 | 0 | 0 | 70 | 0 | 70 | 13 | 12 | 1,200 |
| **36** | 30 | 25 | 0 | 0 | 12 | 1,200 | 30 | 50 | 70 | 13 | 12 | 1,200 |
